# User perspectives on the Swedish Maternal Health Care Register

**DOI:** 10.1186/s12913-014-0613-2

**Published:** 2014-12-10

**Authors:** Kerstin Petersson, Margareta Persson, Marie Lindkvist, Margareta Hammarström, Ingrid Haglund, Carin Nilses, Yvonne Skogsdal, Ingrid Mogren

**Affiliations:** Department of Clinical Sciences, Obstetrics and Gynecology, Umeå University, Umeå, Sweden; Dalarna University, School of Health and Social Studies, Falun, Sweden; Umeå School of Business and Economics, Department of Statistics, Umeå University, Umeå, Sweden; Department of Clinical Science and Education, Södersjukhuset, Karolinska Institutet, Stockholm, Sweden; Primary Health Care, Parental and Child Health Care, Östersund, Sweden; Department of Research and Development, Västernorrland County Council, Sundsvall, Sweden; Primary Health Care, Maternal Health Care Unit, Örebro, Sweden

## Abstract

**Background:**

Established in 1999, the Swedish Maternal Health Care Register (MHCR) collects data on pregnancy, birth, and the postpartum period for most pregnant women in Sweden. Antenatal care (ANC) midwives manually enter data into the Web-application that is designed for MHCR. The aim of this study was to investigate midwives’ experiences, opinions and use of the MHCR.

**Method:**

A national, cross-sectional, questionnaire survey, addressing all Swedish midwives working in ANC, was conducted January to March 2012. The questionnaire included demographic data, preformed statements with six response options ranging from zero to five (0 = totally disagree and 5 = totally agree), and opportunities to add information or further clarification in the form of free text comments. Parametric and non-parametric methods and logistic regression analyses were applied, and content analysis was used for free text comments.

**Results:**

The estimated response rate was 53.1%. Most participants were positive towards the Web-application and the included variables in the MHCR. Midwives exclusively engaged in patient-related work tasks perceived the register as burdensome (70.3%) and 44.2% questioned the benefit of the register. The corresponding figures for midwives also engaged in administrative supervision were 37.8% and 18.5%, respectively. Direct electronic transfer of data from the medical records to the MHCR was emphasised as significant future improvement. In addition, the midwives suggested that new variables of interest should be included in the MHCR – e.g., infertility, outcomes of previous pregnancy and birth, and complications of the index pregnancy.

**Conclusions:**

In general, the MHCR was valued positively, although perceived as burdensome. Direct electronic transfer of data from the medical records to the MHCR is a prioritized issue to facilitate the working situation for midwives. Finally, the data suggest that the MHCR is an underused source for operational planning and quality assessment in local ANC centres.

**Electronic supplementary material:**

The online version of this article (doi:10.1186/s12913-014-0613-2) contains supplementary material, which is available to authorized users.

## Background

### Antenatal care

Almost all pregnant women in Sweden attend antenatal care (ANC), a health service free of charge for pregnant women [1]. National and local guidelines regulate the health care provided at both public and private ANC centres. Midwives working in ANC are responsible for surveillance of pregnant women in accordance with current guidelines, and providing referral for obstetric assessment when potential complications are detected. In addition to surveillance of pregnancies, ANC midwives provide parental support, counselling on family planning, and screening for cervical cancer [1]. Furthermore, midwives manage different administrative systems related to the provided health care, such as registration of data in electronic medical records. Swedish ANC centres are mainly organized within primary health care, and the majority of the ANC centres monitor up to 200 pregnant women per year (personal communication). The mean number of pregnant women requiring health care per full time employed midwife and year is estimated to be 85, a figure that has been stable during the last decade (personal communication). The midwives’ work tasks at ANC centres in Sweden does not include birth assistance.

Sweden is divided into 21 counties, including 43 maternal health care areas. The number of ANC centres differs in each maternal health care area depending on the area’s population. For each maternal health care area, a senior consultant obstetrician and a senior consultant midwife provide local medical guidelines based on national recommendations and aspects of local health care organization [1].

### Health data registers and quality registers in Sweden

The Swedish National Board of Health and Welfare (NBHW) administer a number of health data registers that monitor the general population. The first register to monitor the general population – the Cause of Death Register – started to collect data in 1952. In later years, the Swedish Cancer Register (1958), the Swedish Patient Register (1968), and the Swedish Medical Birth Register (1973) began collecting data. All health data registers are regulated by the Health Data Law in the Swedish Code of Statutes (1998:543), a law that requires the health care system and patients to provide these registers the requested information [2].

During the last decades, an increasing number (N = 79 at present; personal communication) of quality registers have been established in Sweden [3]. All national quality registers are monitored and approved for governmental financing by an Executive Committee in a central organization of the Swedish counties. All quality registers have been initiated by Swedish health care professional associations, in different medical areas of interest. Quality registers collect data on patient characteristics, diagnoses, medical measures and interventions, and health outcomes. Both health data registers and quality registers use the personal identification number each Swedish citizen is given, allowing for the identification of each patient if the need arises [4]. This type of identification system (health data systems containing personal information) requires secure protocols such as a secure login system where each quality register user identifies himself or herself using an individual code [2]. In contrast to the health data registers, patients are not legally compelled to provide data for quality registers, so all patients are to be informed about the quality register and have an opportunity to withhold their data from the MHCR. Quality registers are regulated by the Swedish code of statutes 2008:355 [2].

### The Swedish Maternal Health Care Register

Established in 1999, the Swedish Maternal Health Care Register (MHCR) is a quality register that collects data on pregnancy, birth, and the postpartum period, including data on the individual pregnant woman and her child [3]. The MHCR aims to provide the health care system with valid data that can be used to improve Swedish health care services. During 2007 to 2009, the MHCR underwent a substantial revision of its variables, and technical solutions. A revised version of the MHCR including a new Web-application was launched on 1 January 2010 [3].

Using a Web-application, ANC midwives manually enter all data into the MHCR. Data-entry is performed on two occasions – early pregnancy and after birth. The first dataset, including 14 variables, is entered when the pregnant woman registers with the ANC centre, usually during the first trimester. This dataset, as reported by the pregnant woman, includes background characteristics such as parity, maternal weight, height, smoking habits, educational level, and self-rated health. The second dataset is entered after birth and includes information on pregnancy, maternal and fetal outcomes, and the postpartum period (e.g., smoking habits during pregnancy, prenatal diagnostics, mode of birth, date of birth, birth weight, occurrence of gestational diabetes mellitus, number of antenatal visits, participation in prenatal education group, and breastfeeding at four weeks post-partum). In total, the MHCR includes 36 variables covering background data, pregnancy, birth, and postpartum outcomes. Although the objective of the MHCR is to include data on all pregnant women who attend ANC centres, the coverage of individual data on pregnant women in the MHCR during 2011 and 2012 was 81% and 85% of all pregnant women, respectively (personal communication).

### Rational for the study

There is no previous study investigating the user perspectives of the MHCR; hence the rationale of this study was to investigate midwives’ experiences, opinions, and use of the MHCR in order to further develop the register.

### Aims

The overall aim of this study was to investigate midwives’ experiences, opinions and use of the Swedish Maternal Health Care Register. Specific aims to explore were: *i*) how midwives experience using the MHCR Web-application for data entry; *ii)* how midwives use MHCR in their daily work; *iii)* how and to what extent MHCR data are utilized in operational planning of health services; and *iv)* user opinions about potential improvement of the MHCR.

## Methods

### Study design and study sample

This national cross-sectional study used a questionnaire survey addressed to all midwives currently working in Swedish ANC centres and who were eligible to conduct data entry or use data from the MHCR. In addition, all senior consultant midwives representing each MHCA were invited to participate in the study with an exception of three senior consultant midwives, as they were authors of this study (KP, IH, and YS). The survey was performed between January and March 2012.

### Questionnaire

A questionnaire was developed including in total 62 items. The questionnaire was divided into the following sections: *i*) background characteristics of participants; *ii*) design of the Web-application; *iii*) data entry of individual data; *iv*) user manual; and *v*) online reports that could be created by users from the MHCR. Section *v* was divided into two more parts; participants exclusively engaged in patient-related work task answered part one and participants who had reported administrative supervision answered part two. The latter were asked to answer additional questions on how data from MHCR were used in their daily administrative work. A majority of the items included in section *ii* through *v* were formulated as preformed statements with six Likert-type scale options (0 = totally disagree and 5 = totally agree). Most of the statements were written to reflect a positive experience (e.g., *“The manual gave me the information I needed*”), although two statements were written to reflect a negative experience, *(“The register is burdensome”* and *“I question the benefit of the register”*). These latter two statements, characterized by a negative expression, were formulated due to the pre-understanding of the authors related to the experiences conveyed by midwives working in ANC centres.

To complement the preformed statements, 13 spaces for free text comments were placed after each sub-section in the questionnaire to provide the participants an opportunity to express whatever opinion they would like or to add further information to their answer related to the topic of the section. Before distributing the final version of the questionnaire, a pilot version was tested among purposively selected ANC midwives (N = 14) working in five different MHCA. This pilot study resulted in one minor modification of one variable in the questionnaire, (Additional file 1: The Maternal Health Care Register – questionnaire).

### Data collection procedures

As an initial step, the first author (KP) sent an e-mail to the senior consultant midwives in all maternal health care areas (N = 43) to ascertain the number of ANC midwives currently employed in each maternal health care area. All senior consultant midwives provided the number of ANC midwives in their maternal health care area; from these numbers, the total number of ANC midwives at the time of the data collection was estimated to be 1863.

An e-mail with information about the purpose of the questionnaire study and the procedure of distribution of the questionnaire to all midwives in each maternal health care area was sent to all senior consultant midwives (N = 43); i.e. approximately one month prior to the start of the national survey. In January 2012, the questionnaire was distributed by e-mail to all senior consultant midwives for further distribution to the midwives working in ANC centres in each maternal health care area. The attached information informed the eligible participants to print out the questionnaire on paper, respond it anonymously by returning it by post mail to the senior consultant midwife in each maternal health care area. Thereafter, each senior consultant midwife sent the collected questionnaires to the first author (KP). By answering the questionnaire, midwives, i.e. participants, were assumed to have given their consent to participate in the study. The procedure for collecting the questionnaires completed by the senior consultant midwives differed from the procedure for collecting the questionnaires completed by ANC midwives as the first author was acquainted to all senior consultant midwives. Therefore, these questionnaires were sent to a secretary to secure non-identification of the sender.

For each maternal health care area, the questionnaire was marked with a unique code representing the specific maternal health care area (No 1–43). The questionnaires specifically delivered to the senior consultant midwives were labelled with the code 44. This procedure of labelling questionnaires responding to maternal health care area enabled the authors to estimate the response rate for each maternal health care area as well as for the group of senior consultant midwives. In total, two reminders were sent by e-mail to eligible participants via the senior consultant midwives of the maternal health care area.

### Study protocol

The authors developed an Excel®-protocol to register the numeric variables and a Word®-protocol for the free text comments. The 13 free text comments in the questionnaire corresponded to 13 sections in the Word®-protocol, where all comments by the participants were noted. Each statement by a participant related to the specific section was labelled in the Word document by the number of the participant and the maternal health care area to which the participant belonged. A secretary working in the project registered all data. After completed registration of numeric data, the Excel-protocol was transformed into SPSS format for further analysis (SPSS, vs. 20).

### Statistics

Before the analysis of the 49 preformed statements, the participants were categorized into three groups related to work characteristics: group A included midwives exclusively engaged in patient-related work; group B included midwives engaged in both patient-related work tasks and administrative supervision; and group C included midwives exclusively engaged in administrative supervision. When calculating differences between the groups with or without administrative supervision work tasks, group B and C were merged, as group C consisted of only 24 participants (2.4%).

Analyses of data were done using parametric and non-parametric methods. The preformed statements were accompanied with Likert-type scale answering options (0 to 5). In the analysis, a summary of the values of 3 to 5 (i.e., values indicating a high grade of agreement) was calculated and presented. Odds ratios (OR) and their 95% confidence intervals were calculated using logistic univariate and multivariate regression analyses.

#### Independent variables

For the regression analysis, the independent variables were dichotomised. Age of participants was divided in two categories: 27 to 49 years and 50 to 69 years. Participants’ work experience as ANC midwives was divided into two categories: 0 to 10 years and 11 years or more. In addition, participants were dichotomised into groups related to work tasks (i.e., participants with patient-related work tasks exclusively and participants with work tasks including part-time or full-time administrative supervision). Participants were also categorized according to how often they entered data in the MHCR: data entry once a week or more often and data entry a few times per month or less often. Finally, the participants were categorized according to whether the midwife worked in a public or private ANC centre.

#### Dependent variables

Each preformed statement was used as a dependent variable in regression analysis and was dichotomized into two groups: 0–2 (indicating less agreement) and 3–5 (indicating higher agreement).

### Analysis of free text comments

The analysis of the free text comments presents a range of opinions and does not provide any true quantitative estimation. All free texts were read and analysed using inductive content analysis [5]. First, all comments were read thoroughly to obtain a sense of the data. Second, all comments were coded to reflect the content of the comment. The codes were collected in coding sheets and then organised by comparing the codes. Third, categories and subcategories were established while organising the data. The categories provided information aimed at increasing understanding and generating knowledge regarding the phenomenon under study (i.e., the ANC midwives’ experiences as users of the MHCR). During the analysis, the findings were discussed until consensus was established. The analysis resulted in five categories with 15 subcategories.

### Ethical approval

The Regional Ethical Board at Umeå University (Umeå, Sweden) approved the study (Dno 2012-44-31 M).

## Results

Table 1 presents characteristics of MHCA and response rates. At the time of the study, we estimated the number of ANC midwives working in Sweden to be 1863 and 989 of these responded to the questionnaire, resulting in an overall response rate of 53.1%. The response rates varied between the different counties, ranging from 21.5% to 77.6%. Stockholm County – the largest county in Sweden and with five MHCA accounting for 25.6% of all births in Sweden – had a response rate of 46.2%. The response rate for the group of senior consultant midwives was 92.5% (37/40).

**Table 1:** **Number of Maternal Health Care Areas (MHCA) and births per county, estimated number of midwives in each county, and response rate per county**

**County** ^**a**^	**No. of MHCA**	**No. of births n (%)** ^**b**^	**No. of midwives n (%)** ^**c**^	**Response rate n (%)** ^**d**^
Stockholm	5	28 932 (25.6)	413 (22.1)	191 (46.2)
Västra Götaland	6	19 279 (17.0)	312 (16.7)	174 (55.8)
Skåne	5	15 672 (13.8)	205 (11.0)	76 (37.1)
Östergötland	3	5 085 (4.5)	73 (3.9)	31 (42.5)
Uppsala	1	4 124 (3.6)	70 (3.8)	42 (60.0)
Jönköping	3	3 911 (3.4)	66 (3.5)	46 (69.7)
Halland	2	3 236 (2.8)	63 (3.4)	39 (61.9)
Örebro	1	3 208 (2.8)	65 (3.5)	44 (67.7)
Sörmland	1	3 010 (2.6)	65 (3.5)	14 (21.5)
Gävleborg	1	2 874 (2.5)	50 (2.7)	32 (64.0)
Västmanland	1	2 852 (2.5)	50 (2.7)	25 (50.0)
Västerbotten	2	2 835 (2.5)	49 (2.6)	38 (77.6)
Dalarna	1	2 819 (2.5)	65 (3.5)	34 (52.3)
Värmland	1	2 793 (2.5)	63 (3.4)	38 (60.3)
Kalmar	2	2 396 (2.1)	54 (2.9)	32 (59.3)
Västernorrland	2	2 382 (2.1)	35 (1.9)	24 (68.6)
Norrbotten	1	2 321 (2.1)	61 (3.3)	17 (27.9)
Kronoberg	2	2 089 (1.8)	36 (1.9)	21 (58.3)
Blekinge	1	1 522 (1.3)	25 (1.3)	17 (68.0)
Jämtland	1	1 271 (1.1)	35 (1.9)	12 (34.3)
Gotland	1	566 (0.5)	8 (0.4)	5 (62.5)
Total	43	113 177 (100)	1863 (100)	989 (100)
SCM^e^			43	37/40^f^ (92.5)

^a^All counties in Sweden are presented (N = 21).

^b^Number of births 2012. Data from “Population in the country, counties and municipalities on 31/12/2012 and Population Change in 2012” [Internet] Statistics Sweden; 2012 (cited2014, February 4) http://www.scb.se/en_/Finding-statistics/Statistics-by-subject-area/Population/Population-composition/Population-statistics/Aktuell-Pong/25795/Yearly-statistics--Municipalities-Counties-and-the-whole-country/Population-in-the-country-counties-and-municipalities-on-31122012-and-Population-Change-in-2012/.

^c^Presented as proportion of all midwives in antenatal care (n = 1863) (personal communication).

^d^Presented as proportion of number of midwives in each county.

^e^Senior Consultant Midwives.

^f^Three Senior Consultant Midwives were excluded from participating in the study since they were authors of this article and did not respond the questionnaire. Thus, the denominator in this calculation is 40.

**Figure 1:**
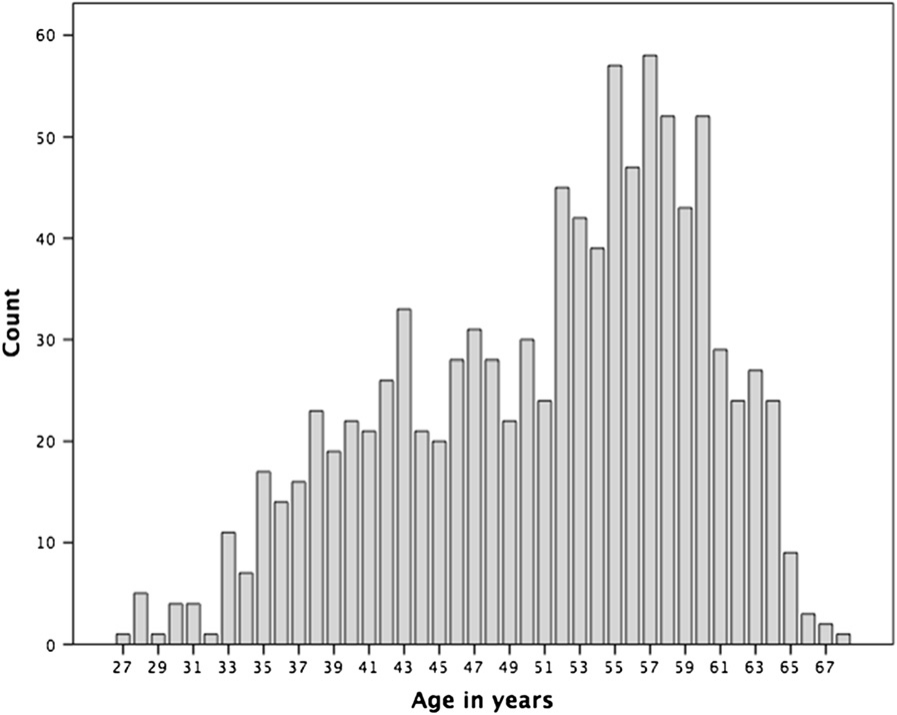
**Participant age distribution.**

Background characteristics of the participants are presented in Table 2 and the age-distribution of participants is presented in Figure 1. The percentage of midwives with patient-related work exclusively (group A) was 89.1%. The percentage of midwives with patient-related work and part-time administrative supervision (group B) was 8.5%. The percentage of midwives with administrative supervision exclusively (group C) was 2.4%. One participant did not report a category. The mean age of all participants was 51.1 years, ranging from 27 to 69 years. The mean age was highest (53.7 years) in group C. The mean age of all midwives in category B and C (53.6 years) was significantly higher than midwives included in category A (50.8 years, p = 0.002). For all participants, the mean number of years of work as a midwife was 21.4 years, whereas the corresponding figure for midwives included in categories B and C were significantly higher (24.8 years, p = 0.001). For all participants, the mean number of years as an ANC midwife was 13.3 years, a finding that suggested that during their career as a midwife the participants had performed other work tasks apart from working in an ANC centre (mean time of 8.1 years). A minor part of the midwives (6.0%) worked less than 0.50 of a full time equivalent while the majority (69.9%) reported a level of employment of 0.75 of a full time equivalent or more. Most midwives (80.4%) entered data in the MHCR at least once a week; this percentage included midwives who entered data daily (7.7%).

**Table 2:** **Characteristics of participating midwives (N = 989) and test of difference between two groups using** ***t*** **-test for numeric variables and Chi-squared test for categorical variables**

**Variable**	**All participating midwives**	**Group A** ^**a**^	**Group B** ^**b**^	**Group C** ^**c**^	**P-value** ^**d**^
		**n = 880 (89.1%)**	**n = 84 (8.5%)**	**n = 24 (2.4%)**	
**Age (yrs) (n, %)**	983 (99.4)	874 (88.0)	84 (8.5)	24 (2.4)	
Mean (SD)	51.1 (8.7)	50.8 (8.9)	53.6 (6.6)	53.7 (6.3)	0.005^e^
Median	53.0	52.5	55.0	55.0	
Range	27-69	27-69	39-64	42-64	
**Work experience as midwife in yrs (n, %)**	941 (95.1)	833 (88.5)	83 (8.8)	24 (2.6)	
Mean (SD)	21.4 (10.5)	21.0 (10.5)	24.7 (9.8)	25.2 (8.8)	0.001^f^
Range	0-44	0-44	4-42	9-40	
**Work experience as midwife in ANC in yrs (n, %)**	906 (99.3)	805 (88.9)	78 (8.6)	22 (2.4)	
Mean (SD)	13.3 (9.3)	13.0 (9.3)	16.0 (8.9)	17.9 (9.0)	<0.001^e^
Range	0-40	0-40	1-36	3-36	
**Working as a midwife in public/private care (n, %)**	977 (98.8)	875 (89.6)	84 (8.6)	17 (1.7)	
Public health care	836 (85.6)	757 (86.5)	63 (75.0)	15 (88.2)	
Private health care	141 (14.3)	118 (13.5)	21 (25.0)	2 (11.8)	
**Level of employment (n,%)**	982 (99.3)	878 (89.4)	84 (8.6)	19 (2.2)	<0.001^g^
<50%^h^	59 (6.0)	37 (4.2)	15 (17.9)	7 (36.8)	
50-75%^i^	237 (24.1)	225 (25.6)	10 (11.9)	1 (5.3)	
>75%^j^	686 (69.9)	616 (70.2)	59 (70.2)	11 (57.9)	
**Registration of data in register (n, %)**	975 (98.6)	873 (89.5)	84 (8.6)	17 (1.7)	<0.001^g^
Daily	75 (7.7)	68 (7.8)	7 (8.3)	0	
Several times a week	585 (59.2)	536 (61.4)	48 (57.1)	0	
Once a week	124 (12.7)	117 (13.4)	6 (7.1)	1 (5.9)	
A few times every month	155 (15.9)	134 (15.3)	19 (22.6)	2 (11.8)	
Less frequent	36 (3.7)	18 (2.1)	4 (4.8)	14 (82.4)	

^a^Group A = Midwives exclusively engaged in patient-related work tasks.

^b^Group B = Midwives engaged in both patient-related work tasks and administrative supervision.

^c^Group C = Midwives exclusively engaged in administrative supervision.

^d^Test of difference between two groups: Midwives with patient-related work tasks exclusively in one group (A) and midwives with patient-related work tasks and supervision and midwives with supervision exclusively in the other group (B, C).

^e^Mann–Whitney.

^f^ *t*-test.

^g^Chi-squared test.

^h^<0.50 of a full time equivalent.

^i^0.50-0.75 of a full time equivalent.

^j^>0.75 of a full time equivalent.

Table 3 presents the results of the preformed statements divided into three main sections: 1) all midwives; 2) midwives executing patient-related work only (Group A); and 3) midwives executing patient-related work tasks and administrative supervision and midwives executing administrative supervision exclusively (Group B and Group C). The response rates for preformed statements included in the three different sections varied as follows: section 1 – 85.0% to 97.7%; section 2 – 85.2% to 95.8%; and section 3 – 73.1% to 91.7%.

**Table 3:** **Response rate and level of agreement on formulated statements related to the Maternal Health Care Register**

	**Response rate** ^**a**^	**Values** ^**b**^						
		**Totally disagree**				**Totally agree**		**Summary of**
	**n (%)**	**0**	**1**	**2**	**3**	**4**	**5**	**value 3 to 5**
**1. Statements responded by all participating midwives (N = 989)**								
It is easy to get an overview	947 (95.8)	9 (0.9)	21 (2.2)	64 (6.8)	261 (27.6)	359 (37.9)	233 (24.6)	853 (90.1)
It is easy to orient myself	948 (95.9)	8 (0.8)	17 (1.8)	60 (6.3)	248 (26.2)	390 (41.1)	225 (23.7)	863 (91.0)
The start page has an appealing layout	866 (87.6)	33 (3.8)	52 (6.0)	134 (15.5)	345 (39.8)	196 (22.6)	106 (12.2)	646 (74.7)
The colours are appealing	844 (85.0)	9 (1.1)	35 (4.1)	99 (11.7)	295 (35.0)	273 (32.3)	133 (15.8)	700 (83.0)
The font is easy to read	943 (95.0)	3 (0.3)	8 (0.8)	33 (3.5)	188 (19.9)	397 (42.1)	314 (33.3)	898 (93.5)
The text is easy to understand	948 (95.9)	3 (0.3)	8 (0.8)	30 (3.2)	184 (19.4)	428 (45.1)	295 (31.1)	907 (95.7)
The font size works well	947 (95.8)	2 (0.2)	2 (0.2)	24 (2.5)	144 (15.2)	402 (42.4)	373 (39.4)	919 (97.0)
I get the information I need about the register	861 (87.1)	19 (2.2)	22 (2.5)	68 (7.9)	236 (27.4)	328 (38.1)	188 (21.8)	752 (87.3)
The Web- application functions well for registration	943 (95.3)	10 (1.1)	14 (1.5)	44 (4.7)	187 (19.8)	420 (44.5)	268 (28.4)	875 (92.8)
**Register manual**								
I have read the manual (proportion of “yes” answer)	493 (51.5)							
The text is easy to understand	462 (93.7)	2 (0.4)	2 (0.4)	14 (3.0)	126 (27.3)	236 (51.2)	82 (17.7)	444 (96.1)
The manual gave me the information I needed	459 (93.1)	3 (0.7)	2 (0.4)	17 (3.7)	121 (26.4)	219 (47.7)	97 (21.1)	437 (92.5)
**Registration of data at first data entry**								
The questions are easy to understand	966 (97.7)	3 (0.3)	2 (0.2)	12 (1.2)	83 (8.6)	379 (39.2)	487 (50.4)	949 (98.2)
The questions come in a logical order	918 (92.8)	4 (0.4)	4 (0.4)	19 (2.1)	117 (12.7)	388 (42.3)	386 (42.0)	891 (97.1)
**Registration of data at second data entry**								
The questions are easy to understand	953 (96.4)	1 (0.1)	2 (0.2)	16 (1.7)	128 (13.4)	425 (44.6)	381 (40.0)	934 (98.0)
The questions come in a logical order	916 (92.6)	2 (0.2)	7 (0.8)	22 (2.4)	132 (14.4)	412 (45.0)	341 (37.2)	885 (96.6)
**2. Statements responded by midwives in group A** ^**c**^ **(n = 880)** ^**d**^								
I regularly access data on pregnant women who visit my clinic	842 (95.7)	481 (57.1)	182 (21.6)	99 (11.8)	55 (6.5)	11 (1.3)	14 (1.7)	80 (9.5)
The register is helpful in my clinical work	750 (85.2)	289 (38.5)	163 (21.7)	118 (15.7)	127 (16.9)	32 (3.6)	21 (2.8)	180 (24.0)
The register is burdensome	843 (95.8)	64 (7.6)	78 (9.3)	105 (12.5)	215 (25.4)	186 (21.9)	197 (23.4)	596 (70.7)
I gain a more coherent picture of the pregnant woman by registering data in the register	809 (91.9)	285 (35.2)	198 (24.5)	131 (16.2)	147 (18.2)	36 (4.4)	12 (1.5)	195 (24.1)
I question the benefit of the register	789 (89.7)	201 (25.5)	148 (18.8)	87 (11.0)	158 (20.0)	83 (10.5)	112 (14.2)	353 (44.7)
**3. Statements responded by midwives in group B** ^**e**^ **(n = 84) and C** ^**f**^ **(n = 24). In total n = 108** ^**d**^								
I regularly access data on pregnant women who visit my clinic	99 (91.7)	20 (20.2)	11 (11.1)	13 (13.1)	22 (22.2)	17 (17.2)	16 (16.2)	55 (55.6)
The register is helpful in my clinical work	85 (78.7)	15 (17.6)	6 (7.1)	11 (12.9)	24 (28.2)	15 (17.6)	14 (16.5)	53 (62.4)
The register is burdensome	98 (90.7)	19 (19.4)	24 (24.5)	18 (18.4)	19 (19.4)	12 (12.2)	6 (6.1)	37 (37.8)
The register is helpful in my administrative work	94 (87.0)	17 (18.1)	7 (7.4)	8 (8.5)	15 (16.0)	28 (29.8)	19 (20.2)	62 (66.0)
I gain a more coherent picture of the pregnant woman by registering data in the register	80 (74.1)	21 (26.3)	8 (10.0)	13 (16.3)	18 (22.5)	14 (17.5)	6 (7.5)	38 (47.5)
I use register data in our operational planning	91 (84.3)	27 (29.7)	11 (12.1)	7 (7.7)	13 (14.3)	22 (24.2)	11 (12.1)	46 (50.5)
I base financial decisions on data from the register	79 (73.1)	37 (46.8)	8 (10.1)	10 (12.7)	14 (17.7)	8 (10.1)	2 (2.5)	24 (30.4)
I use register data to describe the burden of care for my clinic	95 (88.0)	24 (25.3)	7 (7.4)	12 (12.6)	13 (13.7)	23 (24.2)	16 (16.8)	52 (54.7)
I use register data to compare my clinic with other levels of health care (regions, counties, Sweden)	96 (88.9)	22 (22.9)	12 (12.5)	12 (12.5)	8 (8.3)	24 (25.0)	18 (18.8)	50 (52.1)
I present register data to my colleagues at the clinic	96 (88.9)	25 (26.0)	10 (10.4)	7 (7.3)	14 (14.6)	20 (20.8)	20 (20.8)	54 (56.3)
I perceive my colleagues as interested in clinic data	90 (83.3)	15 (16.7)	3 (3.3)	12 (13.3)	21 (23.3)	24 (26.7)	15 (16.7)	60 (66.7)
I provide register data to my supervisors	95 (88.0)	32 (33.7)	11 (11.6)	8 (8.4)	9 (9.3)	19 (20.0)	16 (16.8)	44 (46.3)
I provide register data for development of health care	84 (77.8)	57 (67.9)	13 (15.5)	7 (8.3)	5 (6.0)	1 (1.2)	1 (1.2)	7 (8.3)
I question the benefit of the register	92 (85.2)	52 (56.5)	19 (20.7)	4 (4.3)	4 (4.3)	3 (3.3)	10 (10.9)	17 (18.5)

^a^Response rate is the number of responding participants divided by 989.

^b^Values on a scale from 0 to 5 where 0 = totally disagree and 5 = totally agree.

^c^Group A included midwives exclusively engaged in patient-related work tasks.

^d^One participant did not fill in type of work executed.

^e^Group B included midwives engaged in both patient-related work tasks and administrative supervision.

^f^Group C included midwives exclusively engaged in administrative supervision.

### Response to preformed statements – all participants

These results are presented in Table 3 (section 1). Overall, the participants expressed relatively high agreement (i.e., summary value of 3 to 5) for most statements. For example, the statement *“It is easy to get an overview”* was graded between 3 and 5 by 90.1%, and the corresponding figure for the statement *“The text is easy to understand”* was 95.7%. The statement *“The start page has an appealing layout”* presented a lower level of high agreement (74.7%). Of all the participants, 51.5% reported that they had read the manual; accordingly, 48.5% had not read the manual. Of those participants who had read the manual, 96.1% (a value between 3 and 5) agreed with the statement *“The text is easy to understand”* and 92.5% (a value between 3 and 5) agreed with the statement *“The manual provided me with the information I needed”.* There were four statements regarding first and second data entry. For the first data entry, the statement *“The questions are easy to understand”* were graded between 3 and 5 by 98.2%. The corresponding figure for the same statement for second data entry was 98.0%.

### Statements – participants in group A

These results are presented in Table 3 (section 2): 70.7% of the midwives executing exclusively patient-related work agreed (a value between 3 and 5) with the statement that *“The register is burdensome”*; 9.5% agreed (a value between 3 and 5) with the statement *“I regularly access data on pregnant women who visit my clinic”*; and 44.7% agreed (a value between 3 and 5) with the statement *“I question the benefit of the register”*.

### Statements – participants in merged group B and C

These results are presented in Table 3 (section 3). Of midwives engaged in administrative supervision to some extent or exclusively, 18.5% had a high agreement (a value between 3 and 5) with the statement *“I question the benefit of the register”*. A majority (55.6%) marked a value between 3 and 5 for the statement *“I regularly access data on pregnant women who visit my clinic”* and 66.0% agreed (a value between 3 and 5) to the statement *“The register is helpful in my administrative work*”. Only 8.3% marked a value between 3 and 5 for the statement *“I provide register data for development of health care”.* The corresponding figure for the statement *“I base financial decisions on data from the register”* was 30.4%.

There were statistically significant differences for participants included in group A in relation to participants included in group B and C for the statements *“The register is helpful in my clinical work*” (p < 0.001; COR = 5.22 CI95% 3.26-8.35) and *“I regularly access data on pregnant women who visit my clinic”* (p < 0.001; COR = 11.79 CI95% 7.43-18.69) (Table 4). Group B and C reported more positive values (i.e., agreed with the statement to a higher extent). There were also statistically significant differences for participants included in group A in relation to participants included in group B and C for the statement *“I question the benefit of the register”* (p < 0.001; COR = 0.29 CI95% 0.16-0.49) (Table 4). A statistical difference was identified for participants in the two age groups (p = 0.007) and for participants in the two groups of work experience as midwives (p = 0.004) for the statement *“The register is helpful in my clinical work*”.

**Table 4:** **Univariate and multivariate regression analysis for high agreement of specified statements in relation to background characteristics**

**Variable**	Statements*							
	***“The register is helpful in my clinical work”***				***“I regularly access data on women who visit my clinic”***			
	**Crude OR**	**CI 95%**	**Adjusted OR**	**Adjusted CI 95%**	**Crude OR**	**CI 95%**	**Adjusted OR**	**Adjusted CI 95%**
**Work characteristics**								
Midwives in group A	1				1			
Midwives in group B and C	5.22	3.26-8.35	4.46^a^	2.72-7.29	11.79	7.43-18.69	11.07^b^	6.83-17.91
**Age (years)**								
27-49	1				1			
50-69	1.55	1.12-2.38	1.15^c^	0.77-1.72	2.02	1.33-3.06	1.30^d^	0.76-2.23
**Organisation**								
Public ANC	1				1			
Private ANC	0.71	0.44-1.13			1.35	0.81-2.22		
**Work years in ANC**								
0-10	1				1			
≥11	1.60	1.16-2.19	1.36^e^	0.93-1.99	1.93	1.31-2.83	1.41^f^	0.86-2.29
**Frequency of data entry**								
Once a week or more often	1				1			
A few times every month or less often	0.70	0.46-1.06			1.07	0.67-1.70		
	***“The register is burdensome”***				***“I question the benefit of the register”***			
	**Crude OR**	**CI 95%**	**Adjusted OR**	**Adjusted CI 95%**	**Crude OR**	**CI 95%**	**Adjusted OR**	**Adjusted CI 95%**
**Work characteristics**								
Midwives in group A	1				1			
Midwives in group B and C	0.26	0.16-0.40			0.29	0.16-0.49	0.29^g^	0.16-0.51
**Age (years)**								
27-49	1				1			
50-69	0.81	0.61-1.07			0.973	0.73-1.28		
**Organisation**								
Public ANC	1				1			
Private ANC	1.00	0.67-1.47			1.70	1.15-2.48	1.79^h^	1.21-2.65
**Work years in ANC**								
0-10	1				1			
≥11	0.80	0.60-1.06			0.90	0.67-1.19		
**Frequency of data entry**								
Once a week or more often	1				1			
A few times every month or less often	0.93	0.65-1.31			1.28	0.91-1.79		

*Statements were dependent variables in calculation, see Methods section.

^a^Crude OR adjusted for age and work years in ANC.

^b^Crude OR adjusted for age and work years.

^c^Crude OR adjusted for work years and work characteristics.

^d^Crude OR adjusted for work characteristics and work years in ANC.

^e^Crude OR adjusted for age and work characteristics.

^f^OR adjusted for age and work characteristics.

^g^Crude OR adjusted for organisation.

^h^Crude OR adjusted for work characteristics.

When adjusting for age and work years as an ANC midwife, the adjusted odds ratio remained highly increased for the statements *“The register is helpful in my clinical work”* (AOR = 4.46 CI95% 2.72-7.29) and *“I regularly access data on women who visit my clinic”* (AOR = 11.07 CI95% 6.83-17.91) (Table 4). Midwives employed in private ANC centres agreed with the statement *“I question the benefit of the register”* in a higher degree in relation to midwives employed in public ANC centres (Table 4). The increased odds ratio remained significant after adjustment for work characteristics (Table 4).

The OR for reporting extraction of data from online reports on pregnant women from the MHCR was much higher (OR = 11.90; CI95% 7.52-18.83) for participants engaged in both patient-related work tasks and administrative supervision (group B and C) compared to midwives exclusively engaged in patient-related work tasks.

### Free text comments

The analysis of the free text comments revealed five categories and their corresponding 15 sub-categories. An overview of the categories and sub-categories are presented in Table 5. A summary of each category, including the content of its sub-categories, is presented below in a condensed form and is illustrated with quotations when applicable.

**Table 5:** **Overview of categories and sub-categories for free text answers**

**Categories**	**Sub-categories**
Duplicating registration	Time consuming registration
	Data available in other sources
	Work task for someone else
Navigating the web-application	Old fashioned layout
	Difficulties operating the system
	Patients’ identity exposed
Understanding the variables	Interpretation of variables
	Redundant variables
	Lack of relevant variables
Needing education on the system	Lack of introduction to the system
	Insufficient user instructions
	Need of continuous information
Use of data in daily work	Questioning the usefulness
	Mapping the situation
	Under-utilized source

#### Duplicating registrations

Data entry was reported as time consuming. The midwives saw the time spent registering data as time that could have been used to encounters with pregnant women in the ANC centres. Furthermore, a sub-set of the same data was registered in the medical record as well as in the MHCR. Hence, the midwives considered these data registrations as redundant. To counter this duplication, the many midwives noted that direct data transfer from medical records to the MHCR would simplify this assignment, giving them more time to actually meet with patients. Participants also stated that they did not find it necessary for a midwife to do this work; they noted that a secretary or other administrational staff could enter data into the MHCR.

*I understand the usefulness of the register but because of too many work tasks, it’s always the register that comes second. When time is limited, you always have to prioritize health care.* (Participant no 585)

#### Navigating the Web-application

Participants considered the layout of the Web-application as somewhat old-fashioned and boring. Midwives expressed both positive and negative experiences navigating the software.

*[The register] is easy to use. A bit boring layout to watch . . . but what does it matter . . . “[The register is] not a major stress issue.* (Participant no 347.)

Participants expressed difficulties using the software when entering data. For example, some found it difficult to manage the data entry of a pregnant woman who had moved from one ANC centre to another between the two data entry occasions. That is, a new midwife was responsible for the health care of the woman and subsequently for the second data entry. Extensive use of the computer mouse at managing the Web-application was reported as a difficulty. Furthermore, pregnant women lacking a Swedish personal number made it difficult to enter data into the system. Participants were also concerned about revealing confidential information to other patients due to the layout of the Web-application as a list of pregnant women managed by the specific midwife was clearly visible on the start page. That is, a pregnant woman could see the names and personal numbers of other pregnant women. This situation was seen as potential compromise of patient privacy.

#### Understanding the variables

Some variables in the MHCR (e.g., educational level, country of origin, and occupation) were perceived as having insufficient number of response alternatives. The variable “self-reported health” was reported as sometimes difficult for both the midwife to understand and for the pregnant women to answer. It was suggested that “self-reported health” should be divided into two questions: one regarding physical health and one regarding psychological health. In addition, some midwives felt that variables related to education, country of birth, self-reported health, and prenatal diagnostics were unnecessary. However, there were a number of suggestions on new variables to be included in the MHCR, (e.g., infertility, in vitro fertilization, inter-current diseases during pregnancy, medical complications during pregnancy and birth, and physical activity during pregnancy).

#### Needing education on the system

Participants reported that there had been an insufficient introduction to the Web-application when it was implemented in 2010. Not being properly introduced to the system aggravated the performance of the data-entry. Other comments related to insufficient instructions in the manual concerning specific situations. Participants also requested continuous update on news in the MHCR.

*It would have been helpful if we had received instructions [when the register started] and after a time of practise had had a follow-up meeting with an opportunity to ask questions.* (Participant no 45)

*I want more information and education on how to use the register.* (Participant no 217)

#### Use of data in daily work

The usefulness of data was questioned, and further, the question on insufficient validity of data was raised. A notion was that the variables included in MHCR did not reflect the content of ANC. Some participants also questioned the existence of the MHCR.

*[I have] often wondered who is using the information [the register] and for what? Level of education is not always important, or to whom is it important?* (Participant no 575)

However, others reported using the data to understand the current situation within their own ANC centre. Additionally, data were used to compare local their ANC centre with regional and national trends. Some participants believed that data included in MHCR were underused, and results for a specific ANC data should be more regularly discussed among colleagues. It was also expressed that the data could be used in operational planning and quality assessment to a further extent.

*Even if I don’t fully use the possibilities the register offers today; that is a possibility and a development for the future.* (Participant no 836)

*I wish that we would use the MHCR to get an overview of “our” pregnant women at our ANC and in comparison with other parts of the country. It has to do with burden of care! When I worked in another MHCA, we used to discuss it [the local results] together with the senior consultant midwife. Here, it is never mentioned. It is a topic only discussed in relation to the economy: if we have made mistakes concerning data entry, resulting in a lower bonus.* (Participant no 40)

## Discussion

The main findings of this study demonstrate that participants in general were positive towards using the MHCR Web-application and reported the variables in MHCR as useful and appropriate. However, the majority of midwives who were exclusively engaged in patient-related work reported data entry into the register as burdensome, and four of ten midwives questioned the benefit of the register. The corresponding figures for midwives engaged in administrative supervision were substantially lower. Direct electronic transfer of data from medical records to the MHCR, was emphasized as a significant future improvement of the MHCR.

The estimated overall response rate (53.1%) was calculated from the number of participants and the estimated number of midwives working in an ANC centre at the time of the study. The midwives reported a mean total work experience of 21.4 years and they had worked in ANC for a mean time of 13.3 years. A majority of the participants (80.4%) reported that they performed data entry in the MHCR once a week or more often. These outcomes showed that the midwives were experienced regarding both ANC and management of the MHCR.

The response rates of the preformed statements were high overall. The responses to the statements regarding orientation and layout of the Web-application and data entry demonstrated high values [3-5] (i.e., positive agreement for all participants). Approximately every second participant reported having read the manual, which might seem a surprisingly low figure.

There were no differences between participants for their evaluation of the register for the statements *“I regularly access data on pregnant women who visit my clinic”, “The register is burdensome”,* and *“I question the benefit of the register”* in relation to background characteristics such as age, work experience as midwives, or frequency of data entry in the MHCR. For these three statements, however, there was a statistical difference between midwives exclusively engaged in patient-related work (group A) and midwives engaged in administrative supervision part-time or full-time (group B and C). There was also a statistical difference between midwives employed in private and public ANC centres for the statement *“I question the benefit of the register”*.

The statements “*I use register data in operational planning*” and *“I use register data to compare my clinic with other levels of health care (regions, counties, Sweden)”* demonstrated the values 3 to 5 for 50.5% and 52.1% of participants, respectively (Table 3). These figures seem relatively low considering that one purpose of the quality register is to be used for quality assessment and operational planning. We therefore conclude that the MHCR currently is an under-utilized source for further development of ANC.

Very few studies investigating user experiences of quality registers have been performed. However, one study reports that Nordic departments of gynaecology are interested in participating in quality assessment register as long as participation does not mean any extra work or costs [6], findings consistent with our study. Furthermore, our participants emphasised that electronic direct transfer of data from medical records to the MHCR would substantially reduce their workload. An Australian study shows that e-health platforms result in improvement to error rates and completion levels [7]. It is plausible that a similar improvement would be seen in the MHCR if implementing electronic direct transfer of data. In a questionnaire survey from 2005 investigating the views of orthopaedic consultants in the then newly launched National Joint Registry, a concern was raised that league tables created from register data would be unreliable for both individual surgeons and for hospitals [8].

### Methodological considerations

One strength of this study is that it addressed all midwives currently working in ANC centres in Sweden, so almost all users of the MHCR were given a chance to express their opinions about the register. Additionally, the participants represented all counties in Sweden. With the help of the senior consultant midwives, two reminder e-mails were sent to all participants. A previous postal questionnaire study investigating the effectiveness of follow-up procedures showed that it is worth sending at least three reminders. However, a third reminder resulted in an increased response rate of only 4.4% [9]. This low number may indicate that any further reminder would not have increased the response rate significantly in our study. The response rates for different statements were high overall. Most preformed statements were answered by more than 85% of the participants.

All authors except one had an engagement in the MHCR combined with work experience from ANC. Additionally, as most of the authors had contributed to writing medical guidelines for the ANC centres, they had experienced many encounters with ANC midwives working throughout Sweden where the experiences of using the MHCR had been discussed. The understanding from these encounters constituted the ground for the statements composed in this study. Thus, the authors of this study had significant experience in the field under study and the pilot version of the questionnaire resulted in only a minor revision. The extensive experience may be regarded as a strength as well as a weakness. Although the familiarity with the topic aided in the construction of the questionnaire, this same familiarity and the authors’ pre-understandings might also have excluded important questions. To address this issue, there was an opportunity of all participants to leave comments on issues that were not addressed by the preformed statements or raised in other questions.

Our study may have some other limitations. The estimated response rate was 53.1%, a relatively low percentage considering the desire to produce representative data. However, the exact number of midwives present in their work place during the data collection period may have differed from the numbers provided by the senior consultant midwives. Some eligible participants may have been on sick leave, holiday, or attending training during the study period. Hence, the actual response rate may be somewhat higher than the calculated rate. The response rates in the counties ranged from 21.5% to 77.6%. The coverage of data in MHCR (2011) for those two counties was 53% and 92%, respectively (personal communication). A Cochrane Review identifying effective strategies to increase response rates to postal and electronic questionnaires shows that “a more interesting topic” for eligible participants significantly increased the response rate in both postal and electronic questionnaires [10]. The response rate in our study may thus indicate the level of engagement with the MHCR and may be a contributing factor to the proneness to respond to the questionnaire. Unfortunately, no official data are available on the number of midwives working in ANC centres or hospitals in Sweden, so the degree of non-participation can only be roughly estimated. Official data from the National Board of Health and Welfare show that the mean age and median age were 49 years [11] and 51 years, respectively (personal communication), for all working midwives (N = 7001) in Swedish health care in November 2011. In our study, the mean age of participants was 51.1 years. Since the mean age of the participants in the present study was fairly close to the official data, we believe most probably that our participants are representative for the group under study. Concerning the free comments provided by the participants, it was not possible to establish whether the midwives conveyed their total range of opinions or just a sub-set of their opinions.

### Implications of this study

The results of this study show several potential improvements for the MHCR in relation to the perspectives of its users. The authors concluded, based on the free comments, that there was a call for education on how to handle the data-entry and how to access local and national data practically from the MHCR. There was also identified a need to provide information on how to use register data in quality assessment and in operational planning. After performing this study, the board of the MHCR have arranged a number of educational meetings during 2012 to 2013 in ANC throughout Sweden, inviting midwives currently working in ANC. Direct electronic transfer of data from medical records to the MHCR should become a prioritized issue to facilitate the working situation of the midwives. When electronic direct transfer of data from medical records to the MHCR will be implemented, several of the new variables suggested by the participants will be included in the MHCR.

## Conclusions

MHCR was generally valued positively, although perceived as burdensome, mainly by midwives working with patient-related work exclusively. Direct electronic transfer of data from medical records to the MHCR is a prioritized issue to facilitate the work situation for midwives. The MHCR is an under-utilized source for operational planning and quality assessment in local ANC.

## Additional files

Additional file 1:
**The Maternal Health Care Register – questionnaire (MHCA code no 1-44).**
