# Supplementary material for: User perspectives on the Swedish Maternal Health Care Register
Source: BMC Health Serv Res. 2014 Dec 10;14:613. doi: 10.1186/s12913-014-0613-2 (PMC4274728; doi:10.1186/s12913-014-0613-2)
Supplement: Additional file 1: — The Maternal Health Care Register – questionnaire (MHCA code no 1-44). [file 12913_2014_613_MOESM1_ESM.doc]

**The Maternal Health Care Register – questionnaire** (MHCA code no 1-44)

Background characteristics

1. Do you work as a:

□ Midwife with exclusively patient-related work tasks

□ Midwife with exclusively administrative supervision

□ Midwife with both administrative supervision and patient-related work tasks

2. Your age (years)?

……………………………………..

3. How long have you been working as a midwife (years)?

……………………………………..

4. How long have you been working as a midwife in antenatal care (years)?

…………………………………………

5. What is your level of employment in relation to full-time employment (as midwife in antenatal care):

□ < 50%

□ 50-75%

□ > 75%

6. What type of clinic do you work at:

□ Antenatal care –private health care

□ Antenatal care –public health care

7. How many pregnant women (mean number) do you meet at the ANC clinic per week?

………………………………………………….

8. I enter data in the Maternal Health Care Register:

□ Daily

□ Several times a week

□ Once a week

□ A few times every month

□ Less often

The following questions refer to the start page of the web-application for the Maternal Health Care Register

**Please consider the following statements and tick your appropriate response alternative!**

9. It is easy to get an overview

0 1 2 3 4 5 no opinion/don´t know

(0=totally disagree and 5= totally agree)

10. It is easy to orient myself

0 1 2 3 4 5 no opinion/don´t know

(0=totally disagree and 5= totally agree)

11. The start page has an appealing layout

0 1 2 3 4 5 no opinion/don´t know

(0=totally disagree and 5= totally agree)

12. The colours are appealing

0 1 2 3 4 5 no opinion/don´t know

(0=totally disagree and 5= totally agree)

13. The font is easy to read

0 1 2 3 4 5 no opinion/don´t know

(0=totally disagree and 5= totally agree)

14. The text is easy to understand

0 1 2 3 4 5 no opinion/don´t know

(0=totally disagree and 5= totally agree)

15. The font size works well

0 1 2 3 4 5 no opinion/don´t know

(0=totally disagree and 5= totally agree)

16. I get the information I need about the register

0 1 2 3 4 5 no opinion/don´t know

(0=totally disagree and 5= totally agree)

17. We appreciate if you have any comments or suggestions on how the start page can be improved:

…………………………………………………………………………………………………………………………………………………

…………………………………………………………………………………………………………………………………………………

The following questions refer to the manual of the Maternal Health Care Register

18. Have you read the user manual of the Maternal Health Care Register?

□ Yes – continue to question no 19.

□ No – continue to question no 22.

**If you have read the manual – please consider the following statements and tick your appropriate response alternative!**

19. The text is easy to understand

0 1 2 3 4 5 no opinion/don´t know

(0=totally disagree and 5= totally agree)

20. The manual gave me the information I needed

0 1 2 3 4 5 no opinion/don´t know

(0=totally disagree and 5= totally agree)

21. We appreciate if you have any comments on how the manual can be improved:

…………………………………………………………………………………………………………………………………………………

…………………………………………………………………………………………………………………………………………………

The following questions refer to entering of data in the web-application of the Maternal Health Care Register

**Please consider the following statements and tick your appropriate response alternative!**

22. The web-application functions well for data-entry

0 1 2 3 4 5 no opinion/don´t know

(0=totally disagree and 5= totally agree)

23. We appreciate if you have any comments or suggestions on how data entry could be improved:

…………………………………………………………………………………………………………………………………………………

…………………………………………………………………………………………………………………………………………………

The following questions refer to the first data entry

**Please consider the following statements and tick your appropriate response alternative!**

24. The questions/items in the first data entry are easy to understand

0 1 2 3 4 5 no opinion/don´t know

(0=totally disagree and 5= totally agree)

25. The questions/items in the first data entry come in a logical order

0 1 2 3 4 5 no opinion/don´t know

(0=totally disagree and 5= totally agree)

26. Grateful for your comments, if any on how the order of the questions/items can be improved:

…………………………………………………………………………………………………………………………………………………

…………………………………………………………………………………………………………………………………………………

27. Are there any other questions you think should be included in the first data entry?

□ Yes – continue to question no 28.

□ No – continue to question no 29.

28. If you think any other questions/items should be included in the first data entry - we appreciate if you could give examples on such questions:

…………………………………………………………………………………………………………………………………………………

…………………………………………………………………………………………………………………………………………………

29. Are there any questions/items in the first data entry that you perceive as unnecessary?

□ Yes – continue to question no 30.

□ No – continue to question no 31.

30. If you perceive any questions/items in the first data entry as unnecessary - we appreciate if you could give examples on such questions:

…………………………………………………………………………………………………………………………………………………

…………………………………………………………………………………………………………………………………………………

The following questions refer to the second data entry

**Please consider the following statements and tick your appropriate response alternative!**

31. The questions/items in the second data entry are easy to understand

0 1 2 3 4 5 no opinion/don´t know

(0=totally disagree and 5= totally agree)

32. The questions/items in the first data entry come in a logical order

0 1 2 3 4 5 no opinion/don´t know

(0=totally disagree and 5= totally agree)

33. Grateful for your comments, if any on how the order of the questions can be improved:

…………………………………………………………………………………………………………………………………………………

…………………………………………………………………………………………………………………………………………………

34. Are there other items that you feel should be included in the second data entry?

□ Yes – continue to question no 35.

□ No – continue to question no 36.

35. If you believe that there are items that should be included in the second data entry, we are grateful if you can give examples of such questions:

…………………………………………………………………………………………………………………………………………………

…………………………………………………………………………………………………………………………………………………

36. Are there any questions/items in the second data entry you perceive as unnecessary?

□ Yes – continue to question no 37.

□ No – continue to question no 38.

37. If you believe that there are items that are unnecessary in the second data entry, we are grateful if you can give examples of such questions

…………………………………………………………………………………………………………………………………………………

…………………………………………………………………………………………………………………………………………………

The following questions refer to the reminder function in the web-application

**Please consider the following statement and tick your appropriate response alternative!**

38. How often do you use the reminder function in the web-application for the Maternal Health Care Register?

□ Regularly

□ Seldom

□ Never

39. Grateful for your comments, if any on how the reminder function can be improved:

…………………………………………………………………………………………………………………………………………………

…………………………………………………………………………………………………………………………………………………

The following questions apply to you with no administrative supervision

If administrative supervision is included in your work, continue to question no 46.

**Please consider the following statements and tick your appropriate response alternative!**

40. I regularly access data on pregnant women who visit my clinic

0 1 2 3 4 5 no opinion/don´t know

(0=totally disagree and 5= totally agree)

41. The register is helpful in my clinical work

0 1 2 3 4 5 no opinion/don´t know

(0=totally disagree and 5= totally agree)

42. The register is burdensome

0 1 2 3 4 5 no opinion/don´t know

(0=totally disagree and 5= totally agree)

43. I gain a more coherent picture of the pregnant woman by registering data in the register

0 1 2 3 4 5 no opinion/don´t know

(0=totally disagree and 5= totally agree)

44. I question the benefit of the register

0 1 2 3 4 5 no opinion/don´t know

(0=totally disagree and 5= totally agree)

45. What items do you find are the most interesting to produce on-line reports on?

…………………………………………………………………………………………………………………………………………………

…………………………………………………………………………………………………………………………………………………

The following questions apply to you who have administrative supervision. If administrative supervision is not included in your work, continue to question no 62.

**Please consider the following statements and tick your appropriate response alternative!**

46. I regularly access data on pregnant women who visit my clinic

0 1 2 3 4 5 no opinion/don´t know

(0=totally disagree and 5= totally agree)

47. The register is helpful in my clinical work

0 1 2 3 4 5 no opinion/don´t know

(0=totally disagree and 5= totally agree)

48. The register is helpful in my administrative work

0 1 2 3 4 5 no opinion/don´t know

(0=totally disagree and 5= totally agree)

49. I find the register burdensome

0 1 2 3 4 5 no opinion/don´t know

(0=totally disagree and 5= totally agree)

50. I gain a more coherent picture of the pregnant woman by registering data in the register

0 1 2 3 4 5 no opinion/don´t know

(0=totally disagree and 5= totally agree)

51. I use register data in operational planning

0 1 2 3 4 5 no opinion/don´t know

(0=totally disagree and 5= totally agree)

52. I base financial decisions on register data

0 1 2 3 4 5 no opinion/don´t know

(0=totally disagree and 5= totally agree)

53. I use register data to describe the burden of care for my clinic

0 1 2 3 4 5 no opinion/don´t know

(0=totally disagree and 5= totally agree)

54. I use register data to compare my clinic with other levels of health care (regions, counties, Sweden)

0 1 2 3 4 5 no opinion/don´t know

(0=totally disagree and 5= totally agree)

55. I present register data to my colleagues at the clinic

0 1 2 3 4 5 no opinion/don´t know

(0=totally disagree and 5= totally agree)

56. I perceive my colleagues as interested in clinic data

0 1 2 3 4 5 no opinion/don´t know

(0=totally disagree and 5= totally agree)

57. I provide register data to my supervisors

0 1 2 3 4 5 no opinion/don´t know

(0=totally disagree and 5= totally agree)

58. I provide register data for development of health care

0 1 2 3 4 5 no opinion/don´t know

(0=totally disagree and 5= totally agree)

59. I provide register data to others (specify to whom)………………………………………………………………….

0 1 2 3 4 5 no opinion/don´t know

(0=totally disagree and 5= totally agree)

60. I question the benefit of the register

0 1 2 3 4 5 no opinion/don´t know

(0=totally disagree and 5= totally agree)

61. If you use register data in another context, please describe briefly!

…………………………………………………………………………………………………………………………………………………

…………………………………………………………………………………………………………………………………………………

The following question allows you to give any further comments on the Maternal Health Care Register

62. Please, give any further comments on the Maternal Health Care Register:

…………………………………………………………………………………………………………………………………………………

…………………………………………………………………………………………………………………………………………………

…………………………………………………………………………………………………………………………………………………

…………………………………………………………………………………………………………………………………………………

…………………………………………………………………………………………………………………………………………………

…………………………………………………………………………………………………………………………………………………

…………………………………………………………………………………………………………………………………………………

…………………………………………………………………………………………………………………………………………………

…………………………………………………………………………………………………………………………………………………

…………………………………………………………………………………………………………………………………………………

…………………………………………………………………………………………………………………………………………………

…………………………………………………………………………………………………………………………………………………

**Thank you for your participation!**
